# Preventive care in general practice among healthy older New South Wales residents

**DOI:** 10.1186/1471-2296-14-83

**Published:** 2013-06-16

**Authors:** Mark F Harris, Fakhrul Md Islam, Bin Jalaludin, Jack Chen, Adrian E Bauman, Elizabeth J Comino

**Affiliations:** 1 Centre for Primary Health Care and Equity, University of New South Wales, Sydney, Australia; 2 School of Public Health and Community Medicine, University of New South Wales, Sydney, Australia; 3 The Simpson Centre for Health Services Research, University of New South Wales, Sydney, Australia; 4 School of Public Health, Sydney University, Sydney, Australia

## Abstract

**Background:**

Despite being at high risk, disadvantaged patients may be less likely to receive preventive care in general practice. This study aimed to explore self-reported preventive care received from general practitioners and the factors associated with this by healthy New South Wales (NSW) residents aged 45–74 years.

**Methods:**

A self-completed questionnaire was sent to 100,000 NSW residents in the 45 and Up cohort study. There was a 60% response rate. After exclusions there were 39,964 participants aged 45–74 years who did not report cardiovascular disease or diabetes. Dichotomised outcome variables were participant report of having had a clinical assessment of their blood pressure (BP), blood cholesterol (BC) or blood glucose (BG), or received advice to eat less high fat food, eat more fruit and vegetables or be more physically active from their GP in the last 12 months. Independent variables included socio-demographic, lifestyle risk factors, health status, access to health care and confidence in self-management.

**Results:**

Most respondents reported having had their BP (90.6%), BC (73.9%) or BG (69.4%) assessed. Fewer reported being given health advice to (a)eat less high fat food (26.6%), (b) eat more fruit and vegetables (15.5%) or (c) do more physical activity (19.9%). The patterns of association were consistent with recognised need: participants who were older, less well educated or overweight were more likely to report clinical assessments; participants who were overseas born, of lower educational attainment, less confident in their own self-management, reported insufficient physical activity or were overweight were more likely to report receiving advice. However current smokers were less likely to report clinical assessments; and rural and older participants were less likely to receive diet or physical activity advice.

**Conclusions:**

This study demonstrated a gap between reported clinical assessments and preventive advice. There was evidence for inverse care for rural participants and smokers, who despite being at higher risk of health problems, were less likely to report receiving preventive care. This suggests the need for greater effort to promote preventive care for these groups in Australian general practice.

## Background

In 2007, preventable chronic diseases comprised 37.8% of premature deaths in Australia [1] which can, in part, be explained by the high prevalence of recognised risk factors including hypertension (30% of the population), dyslipidaemia (50% of the population), inadequate fruit and vegetable consumption (70% of the population), insufficient physical activity (54% of the population), and overweight or obesity (62% of the population) [2]. As most people attend general practice, this is a potential setting for opportunistic preventive care [3]. A variety of behavioural interventions have been demonstrated to modify patient behaviour and physiological risk factors, especially for those at high risk [4-6]. Preventive care has been translated into guidelines which are disseminated to general practitioners (GPs) [7]. However, there are major barriers to assessment, management, and follow up of patients with these risk factors at patient, provider, service and system levels [8-10].

Furthermore the distribution of risk is not equal. Socioeconomically disadvantaged groups suffer a 20% higher burden of chronic disease (cardiovascular disease, diabetes, respiratory disease and arthritis) and risk factors for these conditions such as hypertension and dyslipidaemia [11]. They are more likely to smoke, be insufficiently active, be overweight and/or obese, and have fewer serves of fruit or vegetables compared to higher socio-economic groups [12]. They are also more likely to experience clustering of these risk factors leading to multiple co-morbidities [13].

Despite their higher risk, there is some evidence that disadvantaged patients may be less likely to receive preventive care [8,14]. General practices in disadvantaged areas may be less likely to provide preventive care due to a variety of structural and organisational factors within general practice including accessibility, time available for consultations, competing demands on work time, and higher GP stress [15]. Patient factors may also contribute to low use of preventive care by disadvantaged groups including lower patient health literacy, self-efficacy and expectations of accessibility and quality of care [16-19]. However there is a lack of population based studies of disparities in preventive care in Australian general practice.

The Social, Economic and Environmental Factors (SEEF) Study was undertaken to provide the first integrated analysis of the impact of social, economic and environmental factors on the health of Australians in mid to later life, in order to identify critical intervention points for preventing disease and ameliorating disadvantage, ill health, and morbidity in older Australians. The SEEF Study is a sub-study of the 45 and Up Study, a large cohort study of NSW residents aged 45 years or more [20]. The aim of this paper is to explore the self-reported receipt of preventive care among healthy participants in the SEEF Study and the association between this and social, economic, and environmental factors.

## Methods

### Study population

The 45 and Up Study is Australia’s largest population-based cohort study of healthy ageing of people aged 45 years and over living in the state of NSW, Australia. The 45 and Up Study recruited 266,848 individuals between 2006 and 2009 using progressive random samples of the Medicare Australia database through which national health is administered [21]. The cohort population is relatively heterogeneous. Compared to other population data collections, although the 45 and Up Study population is better educated and more likely to be married and employed, groups with other characteristics are well represented in the sample.

Participants provided written consent for long-term follow-up through repeat questionnaires and linkage of their data to multiple health-related datasets. In 2010, the SEEF Study self-completed questionnaire (available from the authors) was distributed to the first 100,000 participants recruited to the 45 and Up study. The questionnaire was piloted with qualitative analysis of participant understanding and factor analysis of the construct validity and reliability. The total number of SEEF Study participants who returned the reply-paid response was 60,404.

### Study sample

Our study sample was limited to 39,964 SEEF Study participants. SEEF Study participants aged 75 years or over (n = 12,234) were excluded because their pattern of preventive care might be influenced by annual health assessments and care plans funded under Medicare for this age group. Similarly, those who reported cardiovascular disease or diabetes (n = 8,206) were excluded because these conditions are complications of the risk factors being studied.

### Outcome variables

The outcome variables were based on aspects of preventive care as defined in the Royal Australian College of General Practitioners (RACGP) guidelines [7]. Data on preventive care were sought by asking respondents to recall GP visits over the past 12 months to determine if they received a blood pressure, cholesterol or blood glucose check or had been told by the their GP to eat fewer high fat or high cholesterol foods, to eat more fruit and vegetables or to be more physically active.

Dichotomous responses (yes/no) to each of these questions were allowed. These questions were based on questions used and validated in previous studies [2,22]. Thus we examined three measures of clinical assessment: blood pressure (BP), blood cholesterol (BC), and blood glucose (BG); and three types of advice: eat less fatty foods (less fat), eat more fruits and vegetables and undertake more physical activity. The recommended frequency for these is between 2–5 years [23]. However patient memory of specific health services is reduced with time and periods of over 12 months are likely to lead to over-reporting of preventive care [24]. Thus a recall period of 12 months was chosen.

### Independent risk factors

The independent variables included socio-demographic characteristics (age, gender, location of residence, country of birth, household income and educational attainment), patient reported lifestyle risk factors (smoking, diet (daily portions of fruit and vegetables), physical activity and weight), health status (a self-reported history of anxiety, depression, high BP or BC), access to primary health care (waiting time to see particular GP or any GP or urgent same day appointment), and confidence in the management of one’s own condition (Table 1). All but the last of these questions were previously used and validated in other research (see Table 1) and were chosen because of their association in previous research and predictions in theory for health service use [2,25-29]. Data on self-reported education, country of birth and height were drawn from the baseline 45 and up survey conducted 2 years previously [20] (Available from https://www.saxinstitute.org.au/our-work/45-up-study/questionnaires/). Body mass index (BMI) and physical activity were classified according to the guidelines of the Australian Institute of Health and Welfare (AIHW) [30]. Remoteness of residence was measured based on the mean score on the Accessibility Remoteness Index of Australia Plus (ARIA+) for the postcode of the participant’s residential address [31].

**Table 1:** Questions used for independent variables

**Participant characteristics**	**Question**	**Response**	**Source**
**Demographic characteristics**			
Gender	What gender are you?	Male, Female	ABS,
			45&Up
Age	What is your date of birth?	dd/mm/yyyy	ABS,
			45&Up
Country of birth*	In which country were you born?	Australia, UK, Ireland, Italy, China, Greece, New Zealand, Germany, Lebanon, Philippines, Netherlands, Vietnam, Malta, Poland, other (please specify)	ABS, 45&Up
Rurality (ARIA+)	What was your most recent previous residential location?	Postcode or Suburb/Town, State/Territory, If overseas, country	45&Up
Socioeconomic status			
Education*	What is the highest qualification you have completed?	No school certificate or other qualifications; School or intermediate certification (or equiv); Higher School or leaving certificate (or equiv); Trade/apprenticeship (e.g. hairdresser, chef); Certificate/diploma (e.g. child care, technician); University degree or higher.	Adapted from ABS; 45&Up
Household income	What is your usual yearly household income before tax from all sources? (please include wages, benefits, pensions, superannuation etc.)	<$5,000; $5-9,999; $10-19,999; $20-29,999; $30-39,999; $40-49,999; $50-59,999; $60-69,999; $70-79,999; $80-89,999; $90-119,999; $120-149,999; $150,000 or more; I would rather not answer this questions	ABS, 45&Up
**Lifestyle factors**			
Smoking status	Have you ever been a regular smoker?	Yes, No	ABS, 45&Up
	Are you a regular smoker NOW?	Yes No	
	About how much do you/did you smoke on average each day?	Cigarettes per day; Pipes and Cigars per day	
Weight	About how much do you weigh?	Kg or Stones and lbs	ABS, 45&Up
Height*	How tall are you without your shoes?	cm or feet and inches	ABS, 45&Up
Physical activity	How many times did you do each of these activities last week?	Walking continuously for at least 10 min (Times last wk)	ABS, 45&Up
		Vigorous physical activity (Times last wk)	
		Moderate physical activity (Times last wk)	
	If you add up all the time you spent doing each activity last week, how much time did you spend altogether doing each type of activity?	Walking continuously for at least 10 min (hrs and mins)	
		Vigorous physical activity (hours and minutes	
		Moderate physical activity (hours and minutes)	
Diet	About how many services of vegetables do you usually eat each day?	Number of services of cooked vegetables each day; Number of services of raw vegetables each day	ABS & 45&up
	About how many serves of fruit or glasses of fruit juice do you usually have each day	Number of serves of fruit each day; Number of glasses of fruit juice each day	
**Health care**			
Health status	In the last month have you been treated for	Anxiety	45&Up
		Depression	
		High blood pressure	
		High Cholesterol	
Access to GP	Thinking of times when you want to see a particular doctor in your practice or medical centre, how quickly do you usually get to see that doctor?	Same day; next day; 2–3 days; 4–5 days; more than 5 days	Adapted from Comm Fund Survey
	Thinking of times when you want to see ANY doctor in your practice or medical centre, how quickly do you usually get to see that doctor?	Same day; next day; 2–3 days; 4–5 days; more than 5 days	
	If you need to see a GP urgently can you normally get seen on the same day?	Yes, No	
Confidence in managing own health	On a scale of 1 to 10, how confident are you that you can do all the things necessary to manage your health on a regular basis?	1 (not at all confident), 2,3,4,5,6,7,8,9,10(totally confident)	

* Data from 45 and Up baseline survey.

*ABS* Australian Bureau of Statistics National Health Survey [32]*45&Up* repeated 45 and Up baseline survey question [29]*Comm Fund Survey* Commonwealth Fund Survey 2005 [33].

### Statistical analysis

We compared the proportions of self-reported clinical assessment and dietary or physical activity advice provided by GP among the participants in our cross-sectional cohort study. The prevalence rates of our dichotomous principal outcomes (clinical assessment and GP advice) were high. Consequently, we estimated relative risk of clinical assessment and advice among socio-demographic and other independent factors for our common outcomes (>10% ). Generalised estimating equations (GEE) models were applied to explore the associations between the frequencies of self-reported preventive care and the independent variables. Crude or unadjusted and adjusted relative risks (RRs) with 95% confidence intervals were estimated from the univariate and multivariate GEE models respectively using binomial distribution with log link function [34]. RRs were adjusted for age, sex, country of birth, remoteness (ARIA+), income and education, using categories as listed in Tables 2 and 3. The RRs were also adjusted for missing values by including additional categories for “missing values” in the model. Six socio-demographic variables (age, sex, education, income, remoteness and place of birth) were entered into the multivariate analysis, regardless of their significance in the univariate analysis, because of their potential importance. All analyses were carried out in SAS version 9.3 (SAS Institute Inc., Cary, NC, USA). All the tests were two-sided and a p-value of less than or equal to 0.05 was considered statistically significant.

**Table 2:** GP clinical assessments and status of health, lifestyle risk factors, accessing to primary health care (PHC) and health self-management, adjusted for socio-demographic characteristics

**Characteristics**		**Clinical assessments**								
			**Blood pressure**			**Cholesterol**			**Glucose**	
**Demographics**		**% BP**	**RR crude (95% CI)**	RR adj. (95% CI)*	**% BC**	**RRcrude (95% CI)**	RR adj. (95% CI)*	**% BG**	**RR crude (95% CI)**	RR adj. (95% CI)*
Gender	Female (†)	90.3	1	1	71.1	1	1	66.1	1	1
	Male	90.9	1.01(1.00, 1.01)	1.00(1.00, 1.01)	78.0	1.10(1.08, 1.11)	1.09(1.08, 1.10)	74.3	1.12(1.11, 1.14)	1.12(1.11, 1.13)
Age group	45-59 (†)	87.2	1	1	67.7	1	1	63.4	1	1
	60-74-	93.9	1.08(1.07, 1.08)	1.08(1.07, 1.09)	80.2	1.19(1.17, 1.20)	1.17(1.16, 1.19)	75.4	1.19(1.17, 1.21)	1.17(1.16, 1.19)
Country of birth	Australia (†)	91.1	1	1	73.9	1	1	.4	1	1
	Overseas	88.6	0.97(0.96, 0.98)	0.97(0.96, 0.98)	74.2	1.00(0.99, 1.02)	0.99(0.97, 1.00)	69.4	1.00(0.98, 1.02)	0.98(0.97, 1.00)
Rurality (ARIA+)	Major City(†)	91.0	1		76.1	1	1	71.3	1	1
	Inner Regional	90.7	1.00(0.99,1.01)	0.99(0.99, 1.00)	72.6	0.95(0.94, 0.97)	0.96(0.95,0. 97)	68.4	0.96(0.94,0.98)	0.96(0.95,0. 98)
	Outer/Remote	90.4	0.99(0.98, 1.00)	0.99(0.98, 1.00)	72.3	0.95(0.93, 0.97)	0.95(0.94, 0.97)	68.3	0.96(0.94, 0.98)	0.96(0.94, 0.97)
Socioeconomic status										
Education	Less than Year 10	92.5	1.04(1.03, 1.05)	1.02(1.01, 1.03)	78.6	1.12(1.09, 1.14)	1.08(1.05, 1.10)	73.6	1.12(1.09, 1.15)	1.08(1.05, 1.11)
	Year 10	91.9	1.03(1.02, 1.04)	1.01(1.00, 1.02)	76.0	1.08(1.06, 1.10)	1.06(1.04, 1.08)	71.5	1.09(1.07, 1.11)	1.08(1.06, 1.10)
	Year 12	90.3	1.01(1.00, 1.02)	1.01(1.00, 1.02)	75.4	1.07(1.05, 1.09)	1.04(1.02, 1.06)	71.5	1.09(1.07, 1.11)	1.05(1.03, 1.07)
	Certificate/diploma	91.0	1.02(1.01, 1.03)	1.02(1.01, 1.03)	74.1	1.05(1.03, 1.07)	1.05(1.03, 1.06)	69.7	1.06(1.04, 1.08)	1.06(1.04, 1.08)
	University (†)	89.1	1	1	70.4	1	1	65.6	1	1
Household income	<$20,000	91.6	1.03(1.02, 1.04)	0.99(0.98, 1.00)	76.3	1.08(1.06, 1.10)	0.99(0.97, 1.01)	71.8	1.08(1.06, 1.11)	0.99(0.96, 1.01)
	$20,000-$39,999	92.0	1.03(1.02, 1.04)	0.99(0.99, 1.00)	76.6	1.08(1.06, 1.10)	0.99(0.97, 1.01)	72.0	1.09(1.06, 1.11)	0.99(0.97, 1.01)
	$40,000-$59,999	90.8	1.02(1.01, 1.03)	0.99(0.98, 1.00)	74.7	1.06(1.04, 1.08)	0.99(0.98, 1.01)	70.6	1.06(1.04, 1.09)	1.00(0.98, 1.02)
	$60,000-$79,999	90.4	1.01(1.00, 1.03)	1.00(0.99,1.01)	73.3	1.04(1.01, 1.06)	0.99(0.97, 1.01)	68.6	1.03(1.01, 1.06)	0.99(0.97, 1.01)
	$80,000+ (†)	89.1	1	1	70.8	1	1	66.4	1	1
Lifestyle risk factors										
Smoking status	Current	85.9	0.95(0.93, 0.97)	0.96(0.94, 0.98)	65.4	0.88(0.86, 0.91)	0.89(0.86, 0.92)	61.3	0.89(0.86, 0.92)	0.89(0.86, 0.92)
	Ex-smoker	91.5	1.01(1.00, 1.02)	1.01(1.00, 1.01)	75.1	1.01(1.00, 1.03)	1.00(0.99, 1.01)	71.2	1.03(1.02, 1.04)	1.01(1.00, 1.02)
	Never smoked (†)	90.5	1	1	74.0	1	1	69.1	1	1
BMI	Underweight	88.9	1.01(1.00, 1.03)	1.00(0.99, 1.02)	70.5	1.02(0.99, 1.04)	1.00(0.97, 1.03)	66.7	1.05(1.02, 1.08)	1.03(1.00, 1.06)
	Normal weight (†)	88.0	1	1	69.3	1	1	63.7	1	1
	Over weight	91.5	1.04(1.03, 1.05)	1.03(1.02, 1.04)	76.0	1.10(1.08, 1.11)	1.07(1.05, 1.08)	71.2	1.12(1.10, 1.14)	1.08(1.06, 1.10)
	Obese	93.8	1.07(1.06, 1.08)	1.05(1.04, 1.06)	78.7	1.14(1.12, 1.15)	1.11(1.09, 1.13)	76.4	1.20(1.18, 1.22)	1.17(1.15, 1.19)
Physical activity	Sedentary	91.4	1.01(0.99, 1.03)	1.00(0.99, 1.02)	71.6	0.96(0.93, 1.00)	0.96(0.93, 0.99)	68.5	0.98(0.95, 1.02)	0.98(0.95, 1.02)
	Insufficient	89.8	0.99(0.98, 1.00)	0.99(0.99, 1.00)	72.8	0.98(0.96, 0.99)	0.98(0.96, 0.99)	68.9	0.99(0.97, 1.01)	0.99(0.97, 1.01)
	Sufficient (†)	90.8	1	1	74.4	1	1	69.6	1	
Adequate vegetable consumption #	Yes (†)	90.6	1	1	73.9	1		69.4	1	
	No	84.4	0.93(0.86, 1.00)	0.93(0.87, 1.00)	69.3	0.94(0.83, 1.05)	0.91(0.81, 1.02)	64.3	0.93(0.81, 1.05)	0.90(0.79, 1.02)
Health status										
Anxiety	Yes (†)	92.7	1.03(1.02, 1.04)	1.03(1.02, 1.04)	75.4	1.02(1.00, 1.04)	1.05(1.03, 1.06)	71.6	1.04(1.02, 1.06)	1.06(1.04, 1.08)
	No	90.3	1	1	73.7	1	1	69.1	1	1
Depression	Yes (†)	92.6	1.03(1.02, 1.03)	1.03(1.03, 1.04)	74.3	1.01(0.99, 1.02)	1.03(1.01, 1.04)	70.0	1.01(0.99, 1.03)	1.04(1.02, 1.05)
	No	90.2	1	1	73.9	1		69.3	1	
High blood pressure	Yes (†)	99.6	1.13(1.12, 1.13)	1.08(1.07, 1.09)	86.7	1.23(1.21, 1.24)	1.17(1.15, 1.18)	80.8	1.21(1.20, 1.23)	1.15(1.14, 1.17)
	No	88.4	1	1	70.8	1	1	66.6	1	1
High cholesterol	Yes (†)	98.3	1.10(1.10, 1.11)	1.06(1.06, 1.07)	95.8	1.36(1.35, 1.37)	1.15(1.14, 1.16)	84.4	1.26(1.24, 1.28)	1.19(1.18, 1.21)
	No	89.3	1	1	70.4	1	1	67.0	1	1
Access to PHC										
Timing of visiting a particular doctor	Same day (†)	90.4	1		74.2	1		70.6	1	1
	Next day	90.7	1.00(0.99, 1.01)	1.00(0.99, 1.01)	74.5	1.00(0.99, 1.02)	1.00(0.99, 1.02)	70.0	0.99(0.97, 1.01)	0.99(0.97, 1.01)
	2-3 days	91.2	1.01(1.00, 1.02)	1.01(1.00, 1.02)	74.6	1.01(0.99, 1.02)	1.02(1.00, 1.04)	69.6	0.99(0.97, 1.00)	1.00(0.98, 1.02)
	4-5 days	91.0	1.01(1.00, 1.02)	1.01(1.00, 1.02)	73.8	0.99(0.97, 1.02)	1.01(0.99, 1.03)	69.1	0.98(0.96, 1.00)	1.00(0.98, 1.02)
	More than 5 days	90.6	1.00(0.99, 1.01)	1.00(1.00, 1.01)	73.4	0.99(0.97, 1.01)	1.01(0.99, 1.03)	68.7	0.97(0.95, 0.99)	1.00(0.98, 1.02)
Timing of visiting any doctor	Same day (†)	91.1	1	1	74.8	1	1	70.5	1	1
	Next day	90.9	1.00(0.99, 1.01)	1.00(0.99, 1.01)	74.0	0.99(0.97, 1.00)	0.99(0.98, 1.00)	69.1	0.98(0.96, 1.00)	0.98(0.96, 1.00)
	2-3 days	90.7	1.00(0.99, 1.01)	1.00(0.99, 1.00)	72.2	0.96(0.95, 0.98)	0.97(0.96, 0.99)	67.4	0.96(0.94, 0.98)	0.96(0.94, 0.98)
	4-5 days	89.5	0.98(0.97, 1.00)	0.98(0.97, 1.00)	73.0	0.98(0.95, 1.00)	0.98(0.95, 1.00)	68.8	0.98(0.95, 1.01)	0.98(0.95, 1.01)
	More than 5 days	89.0	0.98(0.96, 0.99)	0.98(0.96, 0.99)	72.9	0.97(0.95, 1.00)	0.97(0.94, 0.99)	68.4	0.97(0.94, 1.00)	0.96(0.93, 0.99)
Visiting GP urgently same day	Yes (†)	91.6	1	1	75.3	1	1	70.9	1	1
	No	89.2	0.97(0.96, 0.98)	0.98(0.97, 0.99)	72.9	0.97(0.95, 0.98)	0.98(0.97,1.00)	68.2	0.96(0.94, 0.98)	0.98(0.96, 0.99)
Self-management										
Confidence in managing own health	Yes (†)	90.5	1		73.4	1	1	68.8	1	1
	No	90.6	1.00(0.99, 1.01)	1.01(1.00, 1.01)	74.3	1.01(1.00, 1.02)	1.02(1.00, 1.03)	69.9	1.02(1.00, 1.03)	1.02(1.01, 1.03)

† Reference category, *Adjusted for gender, age, remoteness, country of birth, education and household income. # Adequate = 5 portions or more per day.

**Table 3:** GP advice on diet and physical activity and status of health, lifestyle risk factors, accessing to primary health care and health self-management, adjusted for socio-demographic characteristics

		**Dietary and physical activities advice**								
**Characteristics**		**Eating less cholesterol diet**				**Eating more fruits & vegetables**		**More physical activity**		
		**% Diet**	**RR crude (95% CI)**	RR adj. (95% CI)*	**% FV**	**RR crude (95% CI)**	RR adj. (95% CI)*	**% PA**	**RR crude (95% CI)**	RR adj. (95% CI)*
Demographics										
Gender	Female (†)	26.0	1	1	12.9	1	1	19.5	1	1
	Male	27.4	1.06(1.02, 1.09)	1.07(1.03, 1.11)	19.3	1.50(1.43, 1.57)	1.56(1.48, 1.63)	20.6	1.06(1.02, 1.10)	1.09(1.05, 1.14)
Age group	45-59 (†)	26.3	1	1	15.5	1	1	21.3	1	1
	60-74-	26.8	1.02(0.99, 1.05)	0.97(0.93, 1.00)	15.6	1.00(0.96, 1.05)	0.86(0.82, 0.91)	18.5	0.87(0.84, 0.90)	0.82(0.78, 0.86)
Country of birth	Australia (†)	26.4	1	1	14.8	1	1	1	1	
	Overseas	27.1	1.03(0.99, 1.07)	1.03(0.99, 1.07)	18.2	1.23(1.17, 1.30)	1.22(1.16, 1.29)	21.3	1.09(1.04, 1.14)	1.09(1.04, 1.14)
Rurality (ARIA+)	Major City(†)	27.6	1	1	16.9	1	1	22.0	1	1
	Inner Regional	25.9	0.94(0.90,0.98)	0.92(0.88,0. 96)	14.7	0.87(0.82,0.93)	0.85(0.80, 0.90)	19.0	0.86(0.82,0.91)	0.85(0.80, 0.89)
	Outer/Remote	26.8	0.97(0.93, 1.02)	0.94(0.90, 0.99)	15.2	0.90(0.84, 0.97)	0.87(0.81, 0.93)	18.3	0.83(0.78, 0.88)	0.81(0.76, 0.86)
Socioeconomic status										
Education	Less than Year 10	31.5	1.34(1.26, 1.43)	1.33(1.24, 1.42)	22.7	1.78(1.64, 1.94)	1.69(1.55, 1.85)	24.6	1.34(1.24, 1.45)	1.39(1.29, 1.51)
	Year 10	28.3	1.21(1.15, 1.27)	1.21(1.15, 1.28)	16.5	1.30(1.21, 1.39)	1.40(1.30, 1.50)	21.1	1.15(1.08, 1.22)	1.22(1.15, 1.30)
	Year 12	28.0	1.20(1.14, 1.26)	1.17(1.12, 1.24)	17.9	1.41(1.31, 1.51)	1.28(1.19, 1.38)	20.3	1.10(1.04, 1.17)	1.11(1.05, 1.18)
	Certificate/diploma	26.3	1.12(1.07, 1.18)	1.12(1.07, 1.18)	14.1	1.11(1.03, 1.18)	1.12(1.04, 1.20)	19.1	1.04(0.98, 1.10)	1.06(1.00, 1.12)
	University (†)	23.4	1	1	12.7	1	1	18.4	1	1
Household income	<$20,000	29.5	1.18(1.11, 1.25)	1.10(1.03, 1.18)	21.6	1.54(1.42, 1.66)	1.49(1.37, 1.61)	23.0	1.15(1.08, 1.24)	1.18(1.10, 1.28)
	$20,000-$39,999	27.2	1.08(1.03, 1.14)	1.04(0.98, 1.09)	15.9	1.13(1.06, 1.21)	1.13(1.05, 1.22)	19.4	0.97(0.92, 1.03)	1.02(0.96, 1.09)
	$40,000-$59,999	27.1	1.08(1.03, 1.14)	1.05(0.99, 1.11)	15.2	1.08(1.01, 1.17)	1.09(1.01, 1.18)	19.3	0.97(0.91, 1.03)	1.01(0.95, 1.08)
	$60,000-$79,999	26.3	1.05(0.99, 1.11)	1.03(0.97, 1.09)	15.0	1.07(0.98, 1.16)	1.07(0.99, 1.16)	19.9	1.00(0.93, 1.07)	1.02(0.95, 1.10)
	$80,000+ (†)	25.1	1	1	14.1	1	1	19.9	1	1
Lifestyle risk factors										
Smoking status	Current	26.4	1.01(0.94, 1.09)	0.96(0.89, 1.04)	17.4	1.16(1.05, 1.28)	0.99(0.90, 1.09)	21.0	1.07(0.98, 1.17)	0.99(0.91, 1.08)
	Ex-smoker	27.5	1.06(1.02, 1.10)	1.04(1.00, 1.07)	16.3	1.09(1.03, 1.14)	1.00(0.95, 1.05)	20.4	1.04(1.00, 1.09)	1.02(0.98, 1.07)
	Never smoked (†)	26.0	1	1	15.0	1	1	19.6	1	1
BMI	Underweight	24.9	1.49(1.39, 1.61)	1.48(1.37, 1.59)	15.6	1.74(1.57, 1.93)	1.70(1.54, 1.88)	18.0	1.91(1.74, 2.11)	1.94(1.77, 2.14)
	Normal weight (†)	16.7	1	1	8.9	1	1	9.4	1	1
	Over weight	27.2	1.63(1.56, 1.71)	1.62(1.55, 1.70)	15.8	1.77(1.65, 1.89)	1.64(1.54, 1.76)	19.4	2.07(1.95, 2.21)	2.09(1.96, 2.22)
	Obese	42.6	2.55(2.44, 2.67)	2.52(2.41, 2.64)	26.2	2.93(2.74, 3.13)	2.76(2.59, 2.95)	39.1	4.17(3.93, 4.42)	4.18(3.94, 4.44)
Physical activity	Sedentary	32.2	1.29(1.19, 1.41)	1.25(1.15, 1.36)	25.2	1.84(1.66, 2.03)	1.69(1.53, 1.86)	35.7	2.21(2.04, 2.39)	2.14(1.97, 2.31)
	Insufficient	31.3	1.26(1.21, 1.31)	1.24(1.19, 1.29)	20.1	1.46(1.39, 1.54)	1.40(1.33, 1.47)	30.3	1.88(1.80, 1.96)	1.84(1.77, 1.92)
	Sufficient (†)	24.9	1	1	13.7	1	1	16.2	1	1
Adequate vegetable consumption #	Yes (†)	26.5	1		15.5	1	1	19.9	1	1
	No	32.0	1.21(0.93, 1.56)	1.11(0.86, 1.43)	35.2	2.27(1.79, 2.89)	1.75(1.40, 2.20)	28.8	1.45(1.10, 1.91)	1.28(0.97, 1.69)
Health status										
Anxiety status	Yes	31.8	1.23(1.17, 1.29)	1.23(1.18, 1.29)	18.8	1.24(1.16, 1.33)	1.27(1.19, 1.36)	27.6	1.45(1.38, 1.53)	1.44(1.36, 1.52)
	No (†)	25.9	1	1	15.1	1	1	19.0	1	1
Depression	Yes	31.6	1.23(1.18, 1.29)	1.24(1.19, 1.29)	18.8	1.26(1.19, 1.33)	1.28(1.20, 1.35)	27.8	1.51(1.44, 1.58)	1.49(1.42, 1.56)
	No (†)	25.6	1	1	15.0	1	1	18.5	1	1
High blood pressure	Yes	35.5	1.46(1.41, 1.51)	1.46(1.41, 1.51)	22.1	1.58(1.50, 1.66)	1.56(1.48, 1.64)	30.3	1.74(1.67, 1.81)	1.80(1.73, 1.88)
	No (†)	24.4	1	1	14.0	1	1	17.4	1	1
High cholesterol	Yes	47.5	2.05(1.98, 2.12)	2.06(1.99, 2.13)	26.6	1.93(1.84, 2.04)	1.90(1.80, 1.99)	29.7	1.62(1.54, 1.70)	1.65(1.57, 1.72)
	No (†)	23.2	1	1	13.8	1	1	18.4	1	1
Access to PHC										
Timing of visiting a particular doctor	Same day (†)	27.1	1		17.5	1	1	20.5	1	
	Next day	27.1	1.00(0.95, 1.05)	1.01(0.96, 1.06)	15.9	0.91(0.85, 0.98)	0.95(0.88, 1.02)	20.0	0.97(0.91, 1.04)	1.00(0.94, 1.07)
	2-3 days	26.5	0.98(0.93, 1.03)	1.00(0.95, 1.05)	15.2	0.87(0.81, 0.93)	0.93(0.87, 0.99)	20.3	0.99(0.93, 1.05)	1.03(0.98, 1.10)
	4-5 days	26.1	0.96(0.91, 1.02)	0.98(0.92, 1.04)	14.3	0.82(0.75, 0.89)	0.87(0.80, 0.95)	19.1	0.93(0.87, 1.00)	0.98(0.91, 1.05)
	More than 5 days	26.5	0.98(0.93, 1.03)	0.99(0.94, 1.05)	14.7	0.84(0.78, 0.91)	0.91(0.85, 0.98)	19.8	0.96(0.91, 1.03)	1.03(0.96, 1.09)
Timing of visiting any doctor	Same day (†)	26.7	1		15.7	1	1	20.0	1	
	Next day	26.6	1.00(0.95, 1.04)	0.99(0.95, 1.03)	15.1	0.96(0.91, 1.02)	0.96(0.90, 1.02)	20.3	1.01(0.96, 1.06)	1.02(0.97, 1.08)
	2-3 days	26.9	1.01(0.96, 1.06)	1.00(0.95, 1.05)	15.7	1.00(0.93, 1.07)	0.98(0.92, 1.05)	20.1	1.00(0.95,1.07)	1.02(0.96, 1.09)
	4-5 days	27.2	1.02(0.94, 1.10)	1.01(0.93, 1.09)	15.0	0.96(0.86, 1.07)	0.94(0.84, 1.05)	20.0	1.00(0.91, 1.10)	1.02(0.93, 1.12)
	More than 5 days	28.4	1.06(0.99, 1.14)	1.04(0.97, 1.12)	17.8	1.13(1.03, 1.25)	1.08(0.98, 1.19)	20.8	1.04(0.95, 1.13)	1.06(0.97(1.16)
Visiting GP urgently same day	Yes (†)	27.2	1	1	16.1	1		20.4	1	1
	No	28.6	1.05(1.01, 1.10)	1.04(1.00, 1.09)	16.2	1.00(0.94, 1.07)	0.99(0.93, 1.05)	22.0	1.08(1.02, 1.13)	1.07(1.02, 1.13)
Self-management										
Confidence in managing own health	Yes (†)	20.0	1	1	10.7	1	1	12.4	1	1
	No	31.5	1.57(1.51, 1.63)	1.57(1.52, 1.63)	19.2	1.79(1.70, 1.89)	1.73(1.64, 1.82)	25.6	2.07(1.97, 2.16)	2.05(1.96, 2.15)

† Reference category, *Adjusted for gender, age, remoteness, country of birth, education and household income. # Adequate 5 or more portions per day.

### Ethics

This study was carried out in compliance with the Helsinki Declaration and approved by the Human Research Ethics Committee of the University of Sydney (10-2009/12187). All participants gave full informed written consent.

## Results

### Sample characteristics

The study sample comprised 39,964 healthy adults aged 45–74 years. Their characteristics are summarised in Table 4. Participants were more likely to be female and aged 60 years or older. About one fifth was overseas born (21.9%), 30.1% had a university education and 40.1% reported a household income of $80,000 or more. Although 58.5% reported being overweight or obese, low rates of smoking (5.4%) and physical inactivity (3.3%) were reported. Their long-term health problems included anxiety (11.1%), depression (15.6%), high BP (19.7%), and high BC (13.9%). Many respondents reported having difficulty getting to see the doctor of their choice with 20.9% having to wait for more than 5 days, although few (5.6%) reported waiting this long to see any doctor, and 18.8% reported not being able to make a same day appointment to see their GP. More than half (57.1%) were not confident in managing their own health.

**Table 4:** Characteristics of 39,964 healthy older NSW residents participating in the SEEF Study stratified by socio-demographic, lifestyle risk factors, health status, access to PHC, and confidence in managing own health

**Participant characteristics**		**Frequency**	
		**Number**		**%**
**Demographic characteristics**				
Gender	Female	23653		59.2
	Male	16311		40.8
Age group	45-59	19889		49.8
	60-74	20075		50.2
Country of birth	Australia	31201		78.1
	Overseas	8763		21.9
Rurality (ARIA+)	Major city	15655		49.1
	Inner regional	9745		30.6
	Outer/Remote	6456		20.3
**Socioeconomic status**				
Education	Less than year 10	2880		7.3
	Year 10	7995		20.2
	Year 12	7360		18.6
	Certificate/diploma	9435		23.8
	University	11909		30.1
Household income	<$20,000	3596		10.3
	$20,000-$39,999	7007		20.1
	$40,000-$59,999	5889		16.9
	$60,000-$79,999	4374		12.6
	$80,000+	13946		40.1
**Lifestyle factors**				
Smoking status	Current	2149		5.4
	Ex-smoker	13396		33.6
	Never smoked	24350		61.0
BMI*	Underweight	2882		7.3
	Normal	13407		34.2
	Overweight	14789		37.7
	Obese	8163		20.8
Physical activity	Sedentary	1287		3.3
	Insufficient	8549		21.8
	Sufficient	29351		74.9
**Health status**				
Anxiety status	Yes	4431		11.1
	No	35533		88.9
Depression	Yes	6227		15.6
	No	33737		84.4
High blood pressure	Yes	7880		19.7
	No	32084		80.3
High cholesterol	Yes	5546		13.9
	No	34418		86.1
Access to **PHC**				
Timing of visiting a particular doctor	Same day	7810		20.0
	Next day	7523		19.3
	2-3 days	10534		27.0
	4-5 days	4992		12.8
	More than 5 days	8168		20.9
Timing of visiting any doctor	Same day	19917		52.1
	Next day	8696		22.8
	2-3 days	5492		14.4
	4-5 days	1938		5.1
	More than 5 days	2153		5.6
Visiting GP urgently same day	Yes	28025		81.2
	No	6498		18.8
**Self-management**				
Confidence in managing own health	Yes	17084		42.9
	No	22757		57.1

BMI*: Underweight (BMI:< 18.5), Normal weight (BMI: 18.5-24.9), Over weight (BMI: 25.0-29.9), Obesity (BMI: 30+) according to AIHW [30].

Note: Percentages do not consistently total to 100% due to missing values.

### Preventive care

Preventive health care is summarised in Table 5. Most participants reported receiving clinical assessments from their GP during the preceding 12 months (BP: 90.6%; BC: 73.9%; and BG: 69.4%). Fewer participants reported receiving dietary or physical activity advice (less fat: 26.6%, fruit/vegetables: 15.5%, and physical activity 19.9%). The associations with receipt of preventive care and the independent risk factors are summarised in Tables 2 and 3.

**Table 5:** Preventive care from a general practitioner in the past 12 months reported by 39,964 healthy older NSW residents

**Preventive care**	**Male**	**Female**	**Total**
	**n (%)**	**n (%)**	**N (%)**
	**n = 16,311**	**n = 23,653**	**n = 39,964**
**In the past 12 months, have you:**			
Received a blood pressure check?	14787(90.9)	21265(90.3)	36052(90.6)
Had your cholesterol checked?	12608(77.9)	16650(71.1)	29258(73.9)
Had a blood test to check your glucose levels?	11958(74.3)	15380(66.1)	27338(69.4)
Been told by your GP to eat fewer high fat or high cholesterol foods?	4405(27.4)	6043(26.0)	10448(26.6)
Been told by your GP to eat more fruit and vegetables?	3101(19.33)	2996(12.9)	6097(15.5)
Been told by your GP to be more physically active?	3312(20.6)	4515(19.5)	7827(19.9)

### Socio-demographic factors

Clinical assessments of cholesterol and glucose by a GP were more commonly reported by male participants, those who were older, or who were less well educated in both univariate and multivariate analysis (Table 2). There was no clear pattern of association between clinical assessment and income or country of birth in the adjusted analyses. Rural participants were less likely to report cholesterol or glucose assessment.

Males were more likely to report receiving dietary or physical activity advice (Tables 3). Older participants were less likely to report receiving dietary or physical activity advice. Participants who were born overseas, who had lower education or who had lower income were more likely to report receipt of dietary or physical activity advice. However rural participants were less likely to report receiving this advice.

### Lifestyle factors

The associations between clinical assessments and advice with lifestyle factors are depicted in Tables 2 and 3. Current smokers were less likely to report receiving clinical assessment (BP, BC or BG) in both univariate and multivariate analyses. Participants who were overweight or obese were more likely to report receiving clinical assessment and much more likely to report receiving dietary and physical activity advice than those who were of normal weight. No statistically significant associations were observed with clinical assessment for participants who were underweight although this group were more likely that the normal weight group to report receiving dietary and physical activity advice. Participants who were sedentary or reported insufficient physical activity were more likely to report receiving advice to eat more fruit and vegetables and to undertake more activity (Tables 2 and 3).

### Health status

Participants’ self-reported health status was significantly associated with their report of preventive care. Participants who reported anxiety or depression, hypertension or high cholesterol were more likely to report both clinical assessments and dietary or physical activity advice (Tables 2 and 3).

### Access to primary care and self-management

Participant report of preventive care was not statistically significantly associated with waiting time to see a particular GP or any GP. Being unable to make an urgent same day appointment with their GP was associated with less frequent clinical assessment but more frequent advice on physical activity. Those who had to wait more than 2 days to see their doctor were less likely to report having had their BC assessed. Participants who reported that they were not confident in managing their own health were more likely to report receipt of dietary or physical activity advice, but there was no statistically significant difference in their frequency of clinical assessments.

## Discussion

This is one of the first studies examining the receipt of preventive care in general practice reported by a large sample of older adults in Australia. The study showed that participants reported high rates of clinical assessment for BP, BC and BG in the previous 12 months. However despite high levels of risk from overweight and obesity and physical inactivity, receipt of dietary advice (eat less fatty food, eat more fruit and vegetables) and physical activity advice was less frequently reported. Respondents in previous study of overweight and obese people in South Australia reported broadly similar frequency of lifestyle advice from their GPs [3].

A notable strength of the study was the size of the sample. Participants were randomly selected to be invited from a population-based register (Medicare). The questions used in the survey were based on previously validated questions and the questionnaire was piloted. A limitation was that the 45 and Up Study sample is not representative of all residents over the age of 45 years in NSW. In this sample, smoking and physical inactivity rates were lower than have been observed in the national or NSW health surveys [2,35]. However, the rates of overweight and obesity were very similar to those reported in the NSW health surveys [2]. Therefore, although caution is needed in generalising the frequency of preventive care to the whole population, associations within this and other similar population cohorts have been shown to be valid in this cohort [36] and other similar population cohorts [37]. Consequently we believe that the reported associations between receipt of preventive care and socio-demographic, health and other factors in the 45 and Up Study sample are valid [20].

Another potential limitation is that the data are based on participant self-report and recall by patients or GP care received over the previous 12 months. For example it is possible that some participants with low educational attainment might have under- or over- recalled preventive care provided over the previous year. It is not possible to estimate this effect. Similarly other research has shown that patients tend to overestimate physical activity and under report weight [28,38]. Twelve months was chosen as the period because of the reliability of patient recall but is not strictly in accord with guidelines which recommend clinical assessments and advice at intervals between 2 and 5 years for low risk patients [23]. The proportion of patients receiving recommended preventive care is likely to be underestimated.

It was not possible to draw inferences about causality or the direction of associations from the cross-sectional data reported here. Measures of health status were included in the multivariate analysis to adjust for their effect on receipt of preventive care in accordance with Anderson’s model of health care utilisation [25]. However, preventive care may also positively influence health status. Further follow-up of the 45 and Up Study respondents in the longitudinal cohort may help untangle whether health status precedes poor preventive care or vice versa.

Of more significance was the pattern of association between a range of socio-demographic, health, and other factors and report of preventive actions by participants’ GPs. Preventive care is most appropriate when it targets those at greater risk or need. This is consistent with the findings that participants who were older, less well educated or were overweight were more likely to have had their BP, BC or BG assessed. It is also consistent with the higher frequency of advice on diet or physical activity for those born overseas, those who had lower education and were less confident in self-management, were engaged in insufficient physical activity or were overweight. We were unable to control for the number of visits and patients who visited their GP more frequently may have been more likely to receive preventive advice.

Conversely the ‘inverse care law’ observes that sometimes those who need care the most may receive less care [39]. The findings that smokers were less likely to be tested for BP, BC or BG, and that rural and older participants were less likely to report having been given diet or physical activity advice might reflect this ‘inverse care’. These associations were independent of the measures of access to primary care or confidence in self-management. This association between smoking and non-receipt of preventive care has also been observed in analysis of claims to Australia’s Health Insurance Commission (“Medicare”) linked to the 45 up study [40]. Further research is needed to understand the reasons for this association. It may be that greater priority was given to smoking cessation or that patients were perceived as less receptive to preventive care (although the association was not significant for advice). Regardless further attention is needed to assessing and managing other risk factors in this group.

This is the first study to demonstrate these patterns of preventive care as recalled by patients in Australian general practice. Broadly it is a positive picture. However, the negative associations were concerning. Smoking is one of the most important risk factors for many conditions, and those who remain smokers may be resistant to preventive advice. Yet their smoking also places them at high risk for cardiovascular events, events which may be attenuated by early management of BP, high lipids, diabetes [41].

Even recognising that the recall period was only 12 months, the frequency of this relatively well educated group of participant reporting that they had been given advice on ways they could change their diet and physical activity levels was low. In qualitative research with GPs, we have found that they are often pessimistic about the effectiveness of advice as well as being constrained by lack of time and options for referral to education programs [10,42]. While acknowledging the difficulties, it is important to recognise the valuable contribution which GPs and other health professionals can make in helping patients to change their lifestyle [43-45].

People with lower educational attainment (and thus likely to be less health literate and confident) need appropriate information to support them to improve their lifestyle. This was more frequently reported by in this study. However this does not guarantee quality. Often the quality of communication and advice for patients with poor health litany is lower [46]. It is thus appropriate that diet and physical activity advice was more likely to be reported by participants having less confidence in managing their own health. We need to ensure advice is understandable and able to be acted upon.

The negative association between rurality and both clinical assessment of BC and BG and diet and physical activity advice is concerning. This negative association has been observed in other studies [47]. This is despite the higher burden of chronic disease and higher prevalence of risk factors in rural populations [48]. It may reflect the increase time pressure on rural GPs coping with increased workloads [49]. It reinforces the case for greater availability of other health professionals including nursing and allied professionals to provide preventive interventions as well new models of primary health in rural Australia [50,51].

Clinical assessment appeared to be relatively well provided, at least for the clinical measures included in this study. However the low frequency of lifestyle advice is concerning, especially as similar findings have been reported in other research [8,52]. Given its high levels of population reach, general practice represents an opportunity to offer preventive interventions. Yet there are significant practitioner (e.g. attitudes and skills), patient (knowledge and pattern of use for reactive care) and system barriers (such as workforce availability and the split between Commonwealth and State governments in managing health). Many organisations have a role in helping to address these barriers. The newly established Medicare Locals have a particularly important role in providing support for improved performance at the practice level.

## Conclusion

This study is unique in its assessment of reported preventive care across a large population-based sample. We found that most participants recalled receiving screening for the physiological risk factors (blood pressure, blood cholesterol and blood glucose). However, fewer participants recalled receiving advice to improve their lifestyle. Generally, preventive assessment and advice was reported being provided to those most in need (those with other morbidities, overweight, and less well educated). However rural participants and those who smoked were less likely to report preventive care measures despite their greater risk of chronic disease. It is important that clinicians recognise smokers as a high risk group that needs not only to be encouraged to quit smoking but also to receive other preventive interventions to reduce their cumulative risk. Greater effort is also needed to improve access to diet and physical activity education in rural areas.

## Competing interests

The authors declare that they have no competing interests.

## Authors’ contributions

MFH is a member of the SEEF Steering group. He conceived this research and contributed to the design and implementation of the SEEF Study. He wrote the initial drafts of the paper. FI is the statistician employed on the project. He undertook the data analysis and preparation of the study tables, and wrote the statistical methodology for the manuscript. BJ is an epidemiologist with expertise in epidemiological and health services research. He contributed to the analysis plan and the writing of the manuscript. JC is a statistician and senior research fellow in the Simpson Centre for Health Services Research. He contributed to the analysis plan and the writing of the manuscript. AEB leads the SEEF study. He led the design and implementation of the SEEF survey and contributed to the writing and review of the manuscript. EC is an epidemiologist with expertise in record linkage and analysis of large data collections. She contributed to the conceptualisation of this study, supervised the implementation of the data analysis, and made a significant contribution to the preparation of this manuscript. All authors read and approved the final manuscript.

## Pre-publication history

The pre-publication history for this paper can be accessed here:

http://www.biomedcentral.com/1471-2296/14/83/prepub
